# Impact of Overlapping Heterogeneous Versus Homogeneous Drug-Eluting Stents on Clinical Outcomes: Should They Be Identical?

**DOI:** 10.3390/jcm15218342

**Published:** 2026-10-26

**Authors:** Kerim Esenboğa, Müge Akbulut, Bilge Nazar Ateş, Halil Gülyiğit, Emre Özerdem, Yakup Yunus Yamantürk, Şeyhmus Atan, Eralp Tutar

**Affiliations:** 1 Department of Cardiology, Ankara University School of Medicine, 06230 Ankara, Türkiye; kerimesenboga@yahoo.com (K.E.); iremuge@yahoo.com (M.A.); bilgenazates@gmail.com (B.N.A.); memreozerdem95@gmail.com (E.Ö.); yyyamanturk@gmail.com (Y.Y.Y.); atanseyhmus@gmail.com (Ş.A.); eralptutar@gmail.com (E.T.); 2 Department of Cardiology, Simav State Hospital, 43500 Kütahya, Türkiye; 3 Department of Cardiology, İzmir Tepecik Training and Research Hospital, 35020 İzmir, Türkiye; 4 Department of Cardiology, Yıldırım Beyazıt University Yenimahalle Training and Research Hospital, 06370 Ankara, Türkiye; 5 Department of Cardiology, TOBB ETÜ Hospital, 06560 Ankara, Türkiye; 6 Department of Cardiology, Kırıkkale State Hospital, 06230 Kırıkkale, Türkiye

**Keywords:** drug-eluting stent, stent overlap, heterogeneous overlap, homogeneous overlap, percutaneous coronary intervention, major adverse cardiac events, coronary artery disease

## Abstract

**Background:** Stent overlapping serves as a crucial strategy in achieving complete coverage of diffuse coronary artery lesions, plaque shifts, and edge dissections. **Objectives:** We aimed to evaluate the long-term clinical outcomes of overlapping heterogeneous drug-eluting stents (DES) in comparison to homogeneous DES. **Methods:** A total of 421 patients treated with overlapping DES were included in the study. The study patients were stratified into two groups according to the type of overlap: homogeneous (same antiproliferative drug and stent platform) and heterogeneous (different drug and/or platform). The primary endpoint was a device-oriented composite of cardiac death, target-vessel myocardial infarction and target lesion revascularization; a broader composite of all-cause death, any repeat revascularization, stent thrombosis, in-stent restenosis and cerebrovascular events (MACE) and its components were secondary endpoints. Propensity-score analysis with inverse probability of treatment weighting (IPTW) was used to account for treatment-selection bias. **Results:** The device-oriented composite endpoint occurred in 9.3% and 7.1% (*p* = 0.60), and MACE in 17.6% and 14.7% (*p* = 0.56) of patients with homogeneous and heterogeneous DES, respectively, with no significant difference in any individual component. After IPTW adjustment, heterogeneous overlap was not associated with an increased risk of MACE (odds ratio: 0.45, 95% confidence interval: 0.23–0.86) or of the device-oriented composite endpoint (0.52, 0.22–1.23). In a multivariable model with clinically prespecified covariates, lower left ventricular ejection fraction and greater total stent length, but not HbA1c, were independently associated with MACE. **Conclusions:** In this retrospective cohort, overlapping heterogeneous DES was not associated with an increased risk of adverse events compared with homogeneous DES, including in patients presenting with acute coronary syndromes; however, the modest sample size and the low number of events preclude conclusions of equivalence, and clinically relevant differences cannot be excluded.

## 1. Introduction

Diffuse long coronary artery lesions constitute nearly 20% of all percutaneous coronary interventions (PCIs) [1]. In most circumstances, complete coverage of such lesions with a single coronary stent may not be feasible, thereby necessitating the overlapping deployment of multiple stents. Overlapping stents may also be required to cover plaque shifts and edge dissections.

In the bare metal stent (BMS) era, overlapping stents represented a therapeutic challenge due to an elevated risk of major adverse cardiac events (MACE) [2,3]. However, with the widespread adoption of drug-eluting stents (DES), concerns regarding stent overlap have substantially diminished, owing to inherent characteristics of DES such as potent suppression of neointimal hyperplasia and late luminal loss [4,5]. Nonetheless, studies investigating DES overlap and associated clinical outcomes have yielded conflicting results [6,7]. Furthermore, there is a lack of data concerning the overlap of heterogeneous DES types regarding long-term clinical outcomes.

The aim of the current study is to compare the long-term clinical outcomes of overlapping heterogeneous DES with those of overlapping homogeneous DES.

## 2. Materials and Methods

### 2.1. Study Population

**Figure 1:**
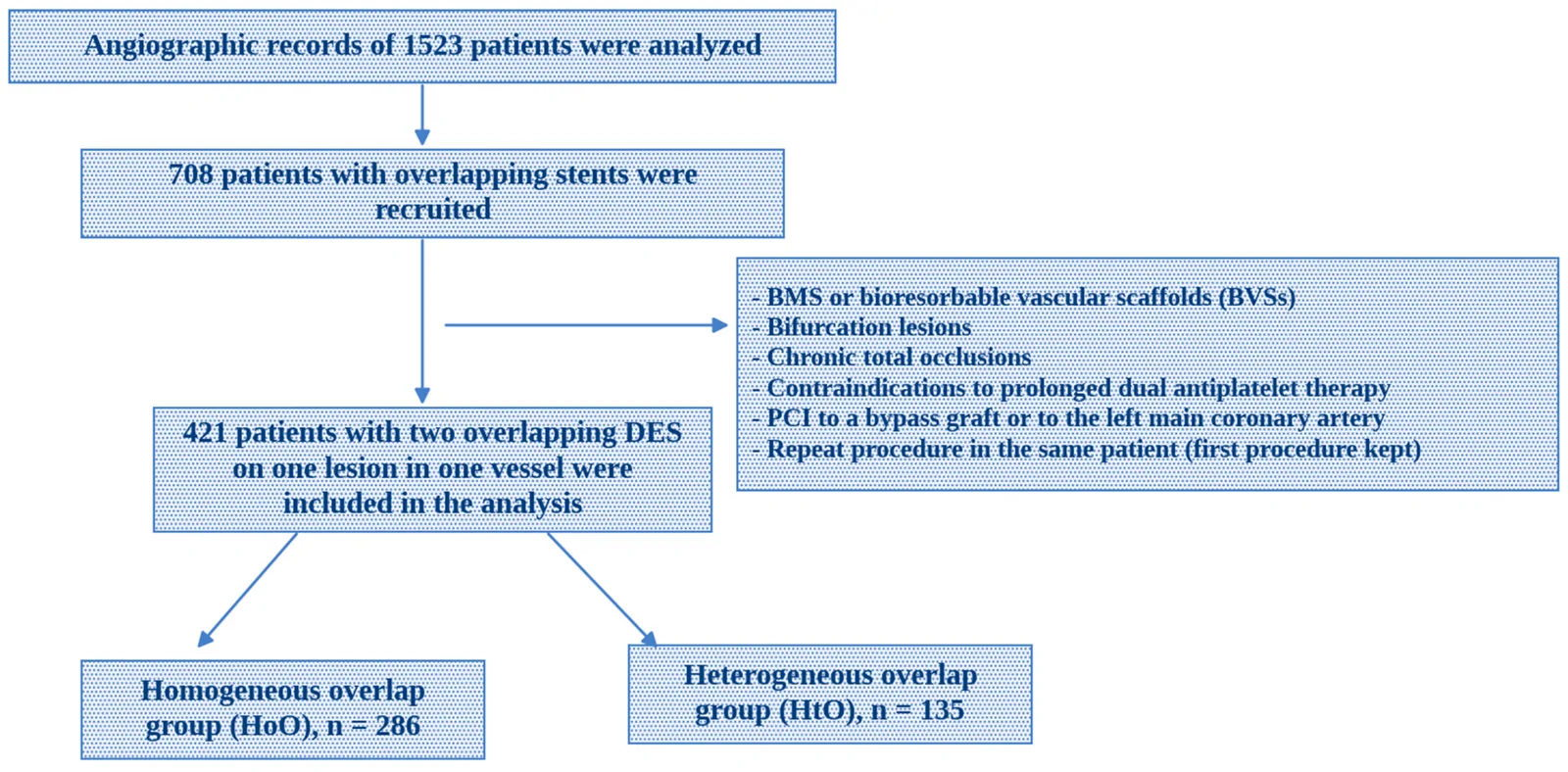
Flow diagram of the study population. BMS, bare metal stent; BVS, bioresorbable vascular scaffold; CTO, chronic total occlusion; DES, drug-eluting stent; HoO, homogeneous overlap; HtO, heterogeneous overlap; PCI, percutaneous coronary intervention.

In this observational study, we retrospectively assessed the angiographic records of a total of 1523 patients undergoing PCI from January 2018 to June 2022 at our institution. Out of these records, we selected 708 patients who received overlapping stents. Patients who were treated with BMS or bioresorbable vascular scaffolds (BVSs) were treated for bifurcation lesions or chronic total occlusions, and had contraindications to prolonged dual antiplatelet therapy were excluded. Patients who had PCI to a bypass graft or to the left main coronary artery were also excluded from the study. For patients who underwent more than one overlapping-stent procedure during the study period, only the first (index) procedure was included. A final total of 421 patients with two overlapping DES on one lesion in one vessel were divided into two groups: homogeneous (HoO) and heterogeneous (HtO) overlap. The study overview is represented in Figure 1.

### 2.2. PCI Procedure

Before the intervention, all patients were given a combination of two anti-platelet drugs: 300 mg aspirin and either 600 mg clopidogrel, 60 mg prasugrel, or 180 mg ticagrelor. Baseline coronary angiography was performed using standard techniques, by using 6 Fr or 7 Fr guiding catheters. Following the decision for PCI, unfractionated heparin was administered intravenously to achieve an activated clotting time of 300 s. The use of glycoprotein IIb/IIIa inhibitors was left to the discretion of the attending operator.

The choice of the stent and overlap type was left to the attending operator’s discretion. Lesion preparation (predilatation), post-dilatation, implantation pressure, and the length of the overlapping segment were likewise left to the operator’s discretion and were not systematically recorded in the procedural database; information on the use of intracoronary imaging (intravascular ultrasound or optical coherence tomography) was not available, and the reasons for choosing a heterogeneous rather than a homogeneous combination (e.g., stent or size availability, operator preference) were not documented. Stent overlap was identified from the angiographic images and procedure reports, and the diameter and length of each implanted stent were recorded. Following stent implantation, all patients were prescribed indefinite aspirin and 6–12 months of P2Y12 inhibitor therapies.

### 2.3. Definitions and Outcomes

Stent overlapping was defined as the presence of ≥2 stents within a single lesion, with an overlapping zone of at least 1 mm. Total stent length was based on the cumulative length of the adjacent stents.

Homogeneous overlap was defined as the overlapping of two stents with the same antiproliferative drug and the same metallic stent platform (cobalt–chromium, platinum–chromium, or stainless steel), and heterogeneous overlap as the overlapping of two stents that differed in antiproliferative drug and/or stent platform. Because the brand of the implanted stents was not recorded in the study database, overlap was classified at the level of drug and platform. The drug-eluting stents in use at our institution during the study period were:

- Biolimus-eluting stent (BES; Terumo, Tokyo, Japan);
- Everolimus-eluting stent (EES; Boston Scientific, Natick, MA, USA);
- Sirolimus-eluting stent (SES; Biotronik, Berlin, Germany);
- Zotarolimus-eluting stent (ZES; Medtronic, Minneapolis, MN, USA);
- Everolimus-eluting stent (EES; Abbott Vascular Inc., Santa Clara, CA, USA);
- Biolimus-eluting stent (BES; Biosensors, Singapore);
- Sirolimus-eluting stent (SES; Terumo, Tokyo, Japan).

The primary endpoint was the device-oriented composite endpoint (DOCE) of cardiac death, target-vessel myocardial infarction, or target lesion revascularization. The secondary endpoint was the broad composite of major adverse cardiac events (MACE) originally prespecified for this study, comprising all-cause death, stent thrombosis, any repeat revascularization, in-stent restenosis, and cerebrovascular events; this composite was retained because it captures the overall patient-level burden of adverse events, but we acknowledge that it is not a standard device-oriented endpoint, and the DOCE is therefore reported as the primary endpoint.

Stent thrombosis was defined according to the Academic Research Consortium (ARC-2) criteria as definite stent thrombosis, i.e., angiographic confirmation of thrombus within the stent or within 5 mm of its edges in the setting of an acute ischemic presentation, or pathological confirmation, and was classified as early (0–30 days), late (31 days to 1 year), or very late (>1 year) [8]. In-stent restenosis was defined as a diameter stenosis of ≥50% within the stented segment or its 5 mm proximal and distal margins on coronary angiography performed for clinical indications; routine angiographic follow-up was not performed. Repeat revascularization was defined as any percutaneous or surgical coronary revascularization after the index procedure, and target lesion revascularization as clinically driven repeat revascularization of the stented segment or its 5 mm margins. Myocardial infarction was defined according to the Fourth Universal Definition of Myocardial Infarction [9] and target-vessel myocardial infarction as myocardial infarction attributable to the target vessel. Cardiac death was defined as any death due to a proximate cardiac cause, procedure-related death, unwitnessed death, or death of unknown cause [8]. Cerebrovascular events were defined as a new focal neurological deficit of vascular origin (stroke or transient ischemic attack) documented in the medical records. All events were identified from hospital records and the national electronic health database and were adjudicated independently by two investigators blinded to the type of overlap; disagreements were resolved by consensus.

Baseline demographic, clinical, laboratory and angiographic data, in-hospital and long-term outcomes were obtained from the medical records by the research team.

The two groups (HoO and HtO) were compared with respect to the DOCE, MACE, and their individual components.

### 2.4. Statistical Analysis

Statistical analyses were conducted with the Statistical Package for Social Sciences (SPSS) software, version 10.0 (IBM Corp., Armonk, NY, USA). Continuous variables were given as means ± standard deviation (SD), whereas categorical ones were given as percentages (%). Continuous variables were analysed using the independent sample *t* test, and categorical variables were analysed with the *χ*^2^ test. Predictors of MACE were examined with multivariable logistic regression using a set of clinically prespecified covariates selected on the basis of prior knowledge rather than univariate significance: age, sex, diabetes mellitus, left ventricular ejection fraction, estimated glomerular filtration rate, acute coronary syndrome presentation, total stent length, and overlap type (heterogeneous versus homogeneous). Because HbA1c was available in only 301 patients (71.5%), diabetes mellitus was used in the primary model, and HbA1c replaced diabetes mellitus in a sensitivity analysis restricted to patients with available values. The number of events limited the models to eight covariates (approximately 7.6 events per variable). Statistical significance was accepted for a two-sided value of *p* < 0.05. Because individual follow-up durations were not available, time-to-event analyses could not be performed; outcomes were therefore compared as cumulative event rates over the follow-up period. Patients without outcome data were excluded from the primary outcome analyses (complete-case analysis). The robustness of the comparison to incomplete follow-up was examined in a sensitivity analysis in which patients with missing outcome data were assumed either to have experienced the event in the heterogeneous group and not in the homogeneous group (worst case) or the reverse (best case). To account for the operator-dependent, non-randomized selection of the overlap strategy, a propensity score for heterogeneous overlap was estimated by multivariable logistic regression including 14 prespecified baseline covariates: age, sex, acute coronary syndrome presentation, hypertension, diabetes mellitus, hyperlipidemia, chronic obstructive pulmonary disease, atrial fibrillation, heart failure, estimated glomerular filtration rate, left ventricular ejection fraction, left anterior descending artery as the target vessel, mean stent diameter, and total stent length. Stabilized inverse probability of treatment weights (IPTW) were derived from the propensity score and truncated at the 99th percentile; covariate balance was assessed with standardized mean differences (SMD), with absolute values below 0.1 considered acceptable. The association between overlap type and each outcome was estimated with IPTW-weighted logistic regression using robust standard errors and is expressed as odds ratios (OR) with 95% confidence intervals (CI). As sensitivity analyses, logistic regression adjusted for the logit of the propensity score and 1:1 nearest-neighbour propensity-score matching without replacement (caliper 0.2 standard deviations of the logit) with cluster-robust standard errors were performed.

## 3. Results

### 3.1. Baseline Characteristics

Of the 421 patients included in the analysis, 244 (58.0%) were female and the mean age was 63.5 ± 11.0 years. A total of 286 patients (67.9%) had homogeneous and 135 (32.1%) had heterogeneous overlapping DES. Patients in the heterogeneous group were less often male (33.3% vs. 46.2%, *p* = 0.013), more often had chronic obstructive pulmonary disease (12.6% vs. 5.2%, *p* = 0.008), and received a smaller first stent (2.76 ± 0.53 vs. 2.85 ± 0.47 mm, *p* = 0.012). The two groups were otherwise similar (Table 1).

In the homogeneous group, the most frequent combinations were two cobalt–chromium everolimus-eluting stents (EES; 60.8%), two platinum–chromium EES (11.5%), two zotarolimus-eluting stents (ZES; 10.8%), two biolimus-eluting stents (BES; 9.4%), and two sirolimus-eluting stents (SES; 7.3%). In the heterogeneous group, the most frequent combinations were a cobalt–chromium EES with a platinum–chromium EES (28.9%), with a BES (23.0%), or with an SES (17.8%). The characteristics of the device classes used are summarized in Table 2, and all overlap combinations are shown in Table 3.

### 3.2. Clinical Outcomes

Clinical follow-up information was obtained from hospital records and the national electronic health database. Because the date of last contact was not systematically recorded for event-free patients, individual follow-up durations could not be calculated; the estimated median follow-up of approximately 1.3 years should therefore be regarded as an approximation. Outcome data were available for 401 of the 421 patients (95.2%); they were unavailable for 14 patients (4.9%) in the HoO group and 6 (4.4%) in the HtO group (*p* = 1.00), and these patients were excluded from the primary outcome analyses and included in the sensitivity analysis described below. The DOCE occurred in 34 patients (8.6%): 25 (9.3%) in the HoO group and 9 (7.1%) in the HtO group (*p* = 0.60). A total of 33 patients (8.4%) died during follow-up, 12 of cardiac causes; target lesion revascularization was required in 26 patients (6.6%), target-vessel myocardial infarction occurred in 2 patients, and 3 patients had a cerebrovascular event. Three cases of stent thrombosis (0.7%) and 15 cases of in-stent restenosis (3.6%) were recorded in the cohort. The broad composite endpoint (MACE) occurred in 67 patients (16.7%): 48 (17.6%) in the HoO group and 19 (14.7%) in the HtO group (*p* = 0.56). None of the individual components differed significantly between the groups (Table 4). Crude odds ratios for heterogeneous versus homogeneous overlap were close to or below unity for the composite endpoints, but all confidence intervals were wide (Table 4); clinically relevant differences in either direction therefore cannot be excluded. In the sensitivity analysis accounting for the 20 patients with missing outcome data, the MACE rates remained similar between groups under the worst-case (HoO 48/286, 16.8% vs. HtO 25/135, 18.5%; *p* = 0.76) and best-case (62/286, 21.7% vs. 19/135, 14.1%; *p* = 0.09) assumptions.

### 3.3. Propensity-Score-Adjusted Analysis

The propensity-score model was fitted in the 400 patients with complete covariate data (c-statistic 0.67). After IPTW, the absolute SMD was below 0.05 for all 14 covariates, indicating adequate balance. After weighting, heterogeneous overlap was not associated with an increased risk of any outcome: the IPTW-weighted OR for heterogeneous versus homogeneous overlap was 0.45 (95% CI 0.23–0.86) for MACE, 0.52 (95% CI 0.22–1.23) for the device-oriented composite endpoint, 0.54 (95% CI 0.23–1.31) for all-cause death, and 0.29 (95% CI 0.09–0.90) for target lesion revascularization (Table 5). The direction of the estimates was consistent in the analysis adjusted for the logit of the propensity score and in the matched analysis (119 pairs), although the confidence intervals included unity (Table 5). Given the modest number of events, these estimates should not be interpreted as evidence of superiority or equivalence.

### 3.4. Predictors of MACE

In the multivariable model with clinically prespecified covariates (n = 380, 61 events), lower left ventricular ejection fraction (OR 0.96 per 1%, 95% CI 0.93–0.98, *p* = 0.001) and greater total stent length (OR 1.03 per mm, 95% CI 1.00–1.06, *p* = 0.023) were independently associated with MACE, whereas overlap type (OR 0.59, 95% CI 0.30–1.14, *p* = 0.12), age, sex, diabetes mellitus, eGFR, and acute coronary syndrome presentation were not (Table 6). In the sensitivity analysis in which HbA1c replaced diabetes mellitus (n = 279, 35 events), HbA1c was not significantly associated with MACE (OR 1.10 per 1%, 95% CI 0.90–1.36, *p* = 0.35), whereas the associations of left ventricular ejection fraction and total stent length persisted (Table 6).

### 3.5. Exploratory Analysis by Overlap Combination

In an exploratory analysis, event rates were examined according to the individual overlap combinations (Table 3). Among the homogeneous combinations, MACE occurred in 21.6% of patients with two cobalt–chromium EES, 13.3% with two platinum–chromium EES, 13.3% with two ZES, 15.0% with two SES, and 4.0% with two BES. Among the heterogeneous combinations with at least 10 patients, MACE rates ranged from 6.9% (BES with cobalt–chromium EES) to 19.4% (cobalt–chromium with platinum–chromium EES), and DOCE rates from 4.2% to 11.8%. No heterogeneous combination showed an apparent excess of events relative to the homogeneous combinations, and MACE rates did not differ significantly across the ten most frequent combinations (*p* = 0.45); however, the number of events within individual combinations was too small for meaningful comparison.

## 4. Discussion

In this real-world cohort, overlapping heterogeneous drug-eluting stents was not associated with a higher incidence of the device-oriented composite endpoint or of MACE and its individual components compared with overlapping homogeneous DES, including all-cause mortality, target lesion revascularization, and cerebrovascular events. These findings were unchanged after accounting for treatment-selection bias with propensity-score weighting and matching, although residual confounding by indication due to unmeasured lesion-, procedure- and operator-related factors cannot be excluded. In a multivariable model with clinically prespecified covariates, lower left ventricular ejection fraction and greater total stent length, rather than HbA1c, were independently associated with MACE.

Stent overlapping is the deployment of two or more stents to treat a single coronary lesion with at least 1 mm of overlapping zone. Excessively long coronary artery lesions constitute the main indication for stent overlapping. The other reasons include edge dissections, thrombus and plaque shifts, and incomplete lesion coverage [10]. At earlier times, stent overlapping was feared by interventional cardiologists due to heightened risk of MACE with overlapping of BMS. However, with the advent of newer generation DES, this fear has been significantly diminished, resulting in an increase of up to 30% in overlapping rates in recent procedures.

Previous investigations have yielded conflicting results regarding DES overlap and adverse clinical outcomes. For instance, the AUTHENTIC trial associated DES with a 2.6-fold increase in definite stent thrombosis [7]. Moreover, a recent study by Coughlan et al. demonstrated that the DES overlap group exhibited a higher incidence of MI within the first 30 days, target vessel revascularization, and stent restenosis, albeit not all-cause mortality [10]. Conversely, a pooled analysis of five clinical trials revealed that the rates of ischemic endpoints at 30 days and 8 months were comparable between overlapping sirolimus-eluting stents (SES) and a single SES [11]. The debate intensifies concerning whether overlapping heterogeneous DES is associated with worse clinical outcomes compared to homogeneous DES overlap. In 2020, Abellán-Huerta et al. reported a higher rate of target vessel revascularization in the homogeneous DES overlap group, hypothesizing that homogeneous DES overlap might exacerbate arterial wall inflammation and delay endothelization more than heterogeneous DES overlap. On the other hand, they noted comparable incidences of cardiac death, myocardial infarction, and stent thrombosis between groups [12]. In contrast, Her et al. reported similar rates of overall MACE, cardiac death, myocardial infarction, and target vessel revascularization between homogeneous and heterogeneous overlap groups [13]. Similarly, we observed no significant differences in the device-oriented composite endpoint, in MACE, or in their components (all-cause mortality, cardiac death, target-vessel myocardial infarction, target lesion revascularization, cerebrovascular events) between the heterogeneous and homogeneous groups. Notably, our study differed from that of Her et al. in terms of a higher proportion of patients with acute coronary syndrome and a smaller average stent diameter (3.18 vs. 2.8 mm). Correspondingly, Liu et al. revealed similar cumulative incidences of MACE at 1 year between homogeneous and heterogeneous DES overlap groups in small (≤2.5 mm) and diffusely diseased (≥30 mm) coronary lesions [14].

During the follow-up period, the device-oriented composite endpoint occurred in 34 (8.6%) and the broad composite endpoint in 67 (16.7%) patients, with comparable incidences between the overlap groups. In their study of patients with acute myocardial infarction, Ahmed et al. [15] found that EES + EES overlap was associated with a lower incidence of MACE than ZES + ZES, SES + SES, and PES + PES. Additionally, Her et al. demonstrated that SES + SES overlap correlated with a reduced need for repeat revascularization compared to PES + PES overlap [13]. Aside from the aforementioned trials, there is a paucity of data comparing the clinical outcomes of different types of second-generation DES overlap. To our knowledge, no other coronary angiography study encompasses such a diverse array of both homogeneous and heterogeneous DES overlap subgroups. While the modest number of patients in these subsets may limit the statistical power of the analysis, the inclusion of such a wide variety of DES overlap subgroups renders our study valuable.

A further consideration is that the heterogeneous group pools combinations that differ in antiproliferative drug, polymer, alloy, and strut design, whereas most homogeneous overlaps (60.8%) consisted of two cobalt–chromium EESs. We consider the pooled comparison clinically meaningful because the question faced by the operator is whether combining two different contemporary DES, whatever their identity, carries an additional risk; biologically, however, specific combinations could behave differently. In the exploratory analysis by combination, no heterogeneous combination showed an apparent excess of events, but the numbers within individual combinations were small, and the study was not designed to compare specific device pairs.

HbA1c was not independently associated with MACE in the multivariable model with clinically prespecified covariates, and HbA1c values were available in only 71.5% of patients, which limits the power of this analysis; any association between glycaemic status and adverse events after overlapping stent implantation therefore remains uncertain. Instead, lower left ventricular ejection fraction and greater total stent length were independently associated with MACE, in line with the established prognostic importance of left ventricular function and with the relation between the length of the stented segment and restenosis [2]. Given the modest number of events, these associations should be regarded as hypothesis-generating.

During the follow-up period, three cases of early stent thrombosis (ST) occurred in our cohort, yielding an incidence of 0.7%, consistent with prior research [13,16]. With only three events, however, the present study is not powered to detect differences in stent thrombosis between the overlap groups, and no conclusion regarding the relative risk of stent thrombosis with heterogeneous overlap can be drawn.

Finally, irrespective of the overlap strategy, secondary prevention after PCI remains essential. Exercise-based cardiac rehabilitation, in particular, has been associated with improved endothelial function and a lower risk of in-stent restenosis after stent implantation [17], although its specific effect in patients with overlapping stents has not been studied.

### Limitations

The present study has several limitations. Firstly, it is a retrospective, non-randomized, single-center study with inherent limitations to the design of the study. Consequently, differences in the amount of overlap may exist between groups. Moreover, procedural characteristics such as the length of the overlapping segment, lesion preparation, post-dilatation, implantation pressure, the degree of calcification, thrombus burden, and the use of intracoronary imaging, as well as the reasons for choosing a heterogeneous rather than a homogeneous combination, were not systematically recorded and could not be compared between the groups, although they may influence outcomes after overlapping stent implantation. Residual confounding by indication therefore cannot be excluded despite propensity-score adjustment. Secondly, intracoronary imaging findings (intravascular ultrasonography or optical coherence tomography) were not available to evaluate the underlying causes of stent thrombosis or restenosis, which represents a significant limitation. Additionally, information regarding smoking status and body mass index was unavailable. Patient-level device (brand) information was not included in the study database; heterogeneity was therefore defined by antiproliferative drug and stent platform, and potential differences between specific devices within the same class could not be examined. HbA1c values were missing in 28.5% of patients, which limited the analysis of glycaemic status as a predictor of adverse events. Furthermore, patterns of in-stent restenosis were not specified, and routine angiographic follow-up data were lacking. Events were adjudicated by two blinded investigators from medical records rather than by an independent clinical events committee. Individual follow-up durations were not available, so the median, range and interquartile range of follow-up could not be reported and Kaplan–Meier and Cox regression analyses could not be performed; event rates were therefore compared with propensity-score-adjusted logistic regression.

## 5. Conclusions

In this retrospective, single-center cohort, overlapping heterogeneous DES was not associated with an increased risk of adverse cardiovascular events compared with homogeneous DES, including in patients presenting with acute coronary syndromes. Given the modest number of events and the observational design, these findings should not be interpreted as evidence of equivalence or proof of safety, and clinically relevant differences cannot be excluded. Lower left ventricular ejection fraction and greater total stent length, rather than glycaemic status, were associated with adverse events. Despite its limitations, we believe that this study offers valuable insights derived from real-world practice. Adequately powered randomized studies are warranted to confirm these findings.

## Figures and Tables

**Table 1:** Baseline demographic, clinical and angiographic characteristics.

Variables	Homogeneous DES (*n* = 286)	Heterogeneous DES (*n* = 135)	*p*-Value
Age (years)	63.9 ± 11.1	62.6 ± 10.6	0.248
Men, n (%)	132 (46.2)	45 (33.3)	0.013 *
Diagnosis, n			0.647
SAP	71	27	
UAP	54	25	
STEMI	68	38	
NSTEMI	92	45	
Co-morbidities, n (%)			
HT	209 (73.1)	99 (73.3)	0.956
DM	131 (45.8)	57 (42.2)	0.490
HL	177 (61.9)	77 (57.0)	0.342
COPD	15 (5.2)	17 (12.6)	0.008 *
AF	13 (4.5)	9 (6.7)	0.361
HF	33 (11.5)	21 (15.6)	0.250
Medications, n (%)			
ACEI	208 (72.7)	101 (75.4)	0.567
ARB	48 (16.8)	19 (14.2)	0.497
BB	256 (89.5)	114 (85.1)	0.191
CCB	67 (23.4)	28 (20.9)	0.563
Statin	281 (98.3)	133 (99.3)	0.669
ASA	283 (99.0)	131 (97.8)	0.389
P2Y12i	286 (100)	134 (100)	—
Laboratory values			
eGFR, mL/min/1.73 m^2^	77.23 ± 19.76	79.39 ± 18.78	0.281
Creatinine, mg/dL	1.03 ± 0.60	1.00 ± 0.36	0.531
Hematocrit, %	40.79 ± 5.48	41.24 ± 5.82	0.456
Hemoglobin, g/dL	13.73 ± 1.94	13.91 ± 2.14	0.386
HbA1c, % †	7.05 ± 1.71	6.88 ± 1.64	0.421
LVEF, %	50.96 ± 10.38	49.56 ± 11.09	0.227
Target vessel, n			0.517
LAD	118	48	
CX	45	22	
RCA	123	65	
Stent			
Mean diameter, mm	2.86 ± 0.44	2.76 ± 0.46	0.040 *
1st stent diameter, mm	2.85 ± 0.50	2.71 ± 0.53	0.012 *
2nd stent diameter, mm	2.88 ± 0.49	2.82 ± 0.48	0.246
1st stent length, mm	27.46 ± 8.11	28.17 ± 7.54	0.379
2nd stent length, mm	24.24 ± 8.78	25.81 ± 8.67	0.087
Total stent length, mm	51.70 ± 11.91	53.99 ± 12.17	0.073

Data are presented as mean ± SD or n (%) unless otherwise stated. † HbA1c was available in 301 patients. * *p* < 0.05 was considered statistically significant. ACEI, angiotensin-converting enzyme inhibitor; AF, atrial fibrillation; ARB, angiotensin receptor blocker; ASA, acetylsalicylic acid; BB, beta-blocker; CCB, calcium channel blocker; COPD, chronic obstructive pulmonary disease; CX, circumflex artery; DES, drug-eluting stent; DM, diabetes mellitus; eGFR, estimated glomerular filtration rate; HbA1c, glycated haemoglobin; HF, heart failure; HL, hyperlipidemia; HT, hypertension; LAD, left anterior descending artery; LVEF, left ventricular ejection fraction; NSTEMI, non-ST-segment elevation myocardial infarction; P2Y12i, P2Y12 inhibitors; RCA, right coronary artery; SAP, stable angina pectoris; STEMI, ST-segment elevation myocardial infarction; UAP, unstable angina pectoris.

**Table 2:** Characteristics of the drug-eluting stent classes in use at our institution during the study period.

Device Class	Platform (Alloy)	Manufacturer	Polymer	Strut Thickness (µm)
EES, CoCr	Cobalt–chromium (L-605)	Abbott Vascular	Durable fluoropolymer (PVDF-HFP/PBMA)	81
EES, PtCr	Platinum–chromium	Boston Scientific	Durable fluoropolymer or bioresorbable abluminal PLGA	74–81
SES, CoCr	Cobalt–chromium (L-605)	Biotronik; Terumo	Bioresorbable PLLA with silicon carbide coating (Biotronik); bioresorbable abluminal PDLLA–PCL (Terumo)	60–80 (Biotronik); 80 (Terumo)
ZES, CoCr	Cobalt–chromium alloy (MP35N) or CoCr shell with Pt–Ir core	Medtronic	Durable BioLinx	81–91
BES, SS	316L stainless steel	Terumo; Biosensors	Bioresorbable abluminal PLA	112–125
SES, SS	316L stainless steel	Not specified (n = 1)	—	—

Characteristics are given according to the manufacturers’ specifications for the devices in the institutional inventory during the study period and are not based on patient-level records; because the specific device was not recorded in the study database for individual patients, the device within a class could not be identified at the patient level. BES, biolimus-eluting stent; CoCr, cobalt–chromium; EES, everolimus-eluting stent; PBMA, poly(n-butyl methacrylate); PDLLA–PCL, poly(D,L-lactide-co-caprolactone); PLA, polylactic acid; PLGA, poly(lactic-co-glycolic acid); PLLA, poly(L-lactic acid); PtCr, platinum–chromium; PVDF-HFP, poly(vinylidene fluoride-co-hexafluoropropylene); SES, sirolimus-eluting stent; SS, stainless steel; ZES, zotarolimus-eluting stent.

**Table 3:** Overlap combinations according to antiproliferative drug and stent platform, with event rates.

Overlap Combination	n (%)	MACE, n/N (%)	DOCE, n/N (%)
Homogeneous overlap (*n* = 286)			
EES (CoCr) + EES (CoCr)	174 (60.8)	36/167 (21.6)	19/166 (11.4)
EES (PtCr) + EES (PtCr)	33 (11.5)	4/30 (13.3)	2/30 (6.7)
ZES (CoCr) + ZES (CoCr)	31 (10.8)	4/30 (13.3)	1/28 (3.6)
BES (SS) + BES (SS)	27 (9.4)	1/25 (4.0)	1/25 (4.0)
SES (CoCr) + SES (CoCr)	21 (7.3)	3/20 (15.0)	2/19 (10.5)
Heterogeneous overlap (*n* = 135)			
EES (CoCr) + EES (PtCr)	39 (28.9)	7/36 (19.4)	4/34 (11.8)
BES (SS) + EES (CoCr)	31 (23.0)	2/29 (6.9)	2/29 (6.9)
EES (CoCr) + SES (CoCr)	24 (17.8)	3/24 (12.5)	1/24 (4.2)
EES (CoCr) + ZES (CoCr)	15 (11.1)	2/14 (14.3)	1/14 (7.1)
BES (SS) + EES (PtCr)	13 (9.6)	2/13 (15.4)	1/12 (8.3)
EES (PtCr) + SES (CoCr)	5 (3.7)	1/5 (20.0)	0/5 (0.0)
BES (SS) + ZES (CoCr)	3 (2.2)	2/3 (66.7)	0/3 (0.0)
EES (PtCr) + ZES (CoCr)	3 (2.2)	0/3 (0.0)	0/3 (0.0)
SES (CoCr) + SES (SS)	1 (0.7)	0/1 (0.0)	0/1 (0.0)
SES (CoCr) + ZES (CoCr)	1 (0.7)	0/1 (0.0)	0/1 (0.0)

Percentages of combinations are calculated within each group; event rates are shown as n/N (%), where N is the number of patients with available outcome data. BES, biolimus-eluting stent; CoCr, cobalt–chromium; EES, everolimus-eluting stent; PtCr, platinum–chromium; DOCE, device-oriented composite endpoint; MACE, major adverse cardiac events; SES, sirolimus-eluting stent; SS, stainless steel; ZES, zotarolimus-eluting stent.

**Table 4:** Clinical outcomes according to the type of overlap.

Outcome, n/N (%)	Homogeneous DES	Heterogeneous DES	Crude OR (95% CI)	*p*-Value
Device-oriented composite endpoint	25/268 (9.3)	9/126 (7.1)	0.75 (0.34–1.65)	0.597
Cardiac death	7/268 (2.6)	5/126 (4.0)	1.54 (0.48–4.95)	0.533
Target-vessel myocardial infarction	2/268 (0.7)	0/126 (0.0)	NE	1.000
Target lesion revascularization	21/268 (7.8)	5/126 (4.0)	0.49 (0.18–1.32)	0.221
Broad composite endpoint (MACE)	48/272 (17.6)	19/129 (14.7)	0.81 (0.45–1.44)	0.556
All-cause death	23/268 (8.6)	10/126 (7.9)	0.92 (0.42–1.99)	0.983
Non-cardiac death	16/268 (6.0)	5/126 (4.0)	0.65 (0.23–1.82)	0.559
Stroke	2/268 (0.7)	1/126 (0.8)	1.06 (0.10–11.85)	1.000

N denotes the number of patients with available outcome data. Crude odds ratios (OR) are for heterogeneous versus homogeneous overlap (reference); NE, not estimable (no events in the heterogeneous group). Stent thrombosis (n = 3) and in-stent restenosis (n = 15) are included in the broad composite endpoint; they are not shown separately by group because the small number of events precludes meaningful comparison. Device-oriented composite endpoint: cardiac death, target-vessel myocardial infarction, or target lesion revascularization. CI, confidence interval; DES, drug-eluting stent; MACE, major adverse cardiac events.

**Table 5:** Propensity-score-adjusted association between heterogeneous overlap and clinical outcomes.

Outcome	HoO, n/N	HtO, n/N	Crude OR (95% CI)	IPTW OR (95% CI)	Matched OR (95% CI)
MACE	46/256	15/124	0.63 (0.34–1.18)	0.45 (0.23–0.86)	0.49 (0.23–1.05)
Device-oriented composite	24/253	8/122	0.67 (0.29–1.54)	0.52 (0.22–1.23)	0.67 (0.25–1.79)
All-cause death	23/253	8/122	0.70 (0.30–1.62)	0.54 (0.23–1.31)	0.55 (0.21–1.42)
Target lesion revascularization	20/253	4/122	0.39 (0.13–1.18)	0.29 (0.09–0.90)	0.47 (0.15–1.50)

Odds ratios are for heterogeneous (HtO) versus homogeneous (HoO) overlap (reference) among the 400 patients with complete covariate data; N denotes patients with available outcome data. The matched analysis comprised 119 propensity-score-matched pairs. Device-oriented composite: cardiac death, target-vessel myocardial infarction, or target lesion revascularization. CI, confidence interval; IPTW, inverse probability of treatment weighting; MACE, major adverse cardiac events; OR, odds ratio.

**Table 6:** Multivariable logistic regression models of predictors of MACE with clinically prespecified covariates.

Variable	Model A: OR (95% CI)	*p*-Value	Model B: OR (95% CI)	*p*-Value
Heterogeneous overlap (vs. homogeneous)	0.59 (0.30–1.14)	0.115	0.43 (0.18–1.03)	0.059
Age (per year)	1.02 (1.00–1.05)	0.098	1.00 (0.96–1.04)	0.945
Male sex	1.15 (0.64–2.07)	0.648	1.22 (0.57–2.65)	0.608
Diabetes mellitus	1.01 (0.56–1.81)	0.970	—	—
HbA1c (per 1%)	—	—	1.10 (0.90–1.36)	0.349
LVEF (per 1%)	0.96 (0.93–0.98)	0.001 *	0.96 (0.93–1.00)	0.032 *
eGFR (per mL/min/1.73 m^2^)	0.99 (0.98–1.01)	0.358	0.99 (0.97–1.01)	0.363
ACS presentation	1.04 (0.49–2.19)	0.922	1.13 (0.39–3.26)	0.826
Total stent length (per mm)	1.03 (1.00–1.06)	0.023 *	1.05 (1.02–1.09)	0.002 *

* *p* < 0.05 was considered statistically significant. Model A: clinically prespecified covariates (n = 380, 61 events; 7.6 events per variable). Model B: sensitivity analysis in which HbA1c replaced diabetes mellitus, restricted to patients with available HbA1c (n = 279, 35 events). ACS, acute coronary syndrome; CI, confidence interval; eGFR, estimated glomerular filtration rate; HbA1c, glycated haemoglobin; LVEF, left ventricular ejection fraction; MACE, major adverse cardiac events; OR, odds ratio.

## Data Availability

The raw data that support the findings of this study are available from the corresponding authors upon reasonable request.

## Abbreviations

The following abbreviations are used in this manuscript:

BES	Biolimus-eluting stent
BMS	Bare metal stent
BVS	Bioresorbable vascular scaffold
CI	Confidence interval
DES	Drug-eluting stent
DOCE	Device-oriented composite endpoint
EES	Everolimus-eluting stent
HbA1c	Glycated haemoglobin
HoO	Homogeneous overlap
HtO	Heterogeneous overlap
IPTW	Inverse probability of treatment weighting
LVEF	Left ventricular ejection fraction
MACE	Major adverse cardiac events
OR	Odds ratio
PCI	Percutaneous coronary intervention
SES	Sirolimus-eluting stent
SMD	Standardized mean difference
ST	Stent thrombosis
TLR	Target lesion revascularization
ZES	Zotarolimus-eluting stent
